# Effects of establishing cultivated grassland on soil organic carbon fractions in a degraded alpine meadow on the Tibetan Plateau

**DOI:** 10.7717/peerj.14012

**Published:** 2022-09-13

**Authors:** Xiang Liu, Xiaotao Huang, Wenping Qin, Xiaoan Li, Zhiwen Ma, Hongxiao Shi, Lanhai Li, Changzhong Li

**Affiliations:** 1 State Key Laboratory of Plateau Ecology and Agriculture, Qinghai University, Xining, China; 2 Key Laboratory of Restoration Ecology for Cold Regions Laboratory in Qinghai, Northwest Institute of Plateau Biology, Chinese Academy of Sciences, Xining, China; 3 Key Laboratory of Adaptation and Evolution of Plateau Biota, Chinese Academy of Sciences, Xining, China; 4 College of Eco-Environmental Engineering, Qinghai University, Xining, China; 5 Institute of Grassland Research, Chinese Academy of Agricultural Sciences, Hohhot, China; 6 Qinghai Academy of Animal and Veterinary Sciences, Qinghai University, Xining, China; 7 State Key Laboratory of Desert and Oasis Ecology, Xinjiang Institute of Ecology and Geography, Chinese Academy of Sciences, Urumqi, China; 8 CAS Research Centre for Ecology and Environment of Central Asia, Urumqi, China

**Keywords:** Soil organic carbon fractions, Biochemical stability, Grassland restoration, Alpine region, C:N ratio

## Abstract

**Background:**

The degradation of alpine meadows has induced substantial losses of soil organic carbon (SOC) on the Tibetan Plateau. A commonly-used method for rehabilitating degraded alpine meadows in this region is establishing cultivated grasslands through sowing seed mixtures, but its impact on the biochemical stability of SOC has remained inadequately explored.

**Methods:**

In this study, a total of 20 composited 0-20 cm soil samples were collected from a heavily degraded alpine meadow (DM) and three adjacent cultivated grasslands established for 3 years (CG3), 12 years (CG12), and 17 years (CG17) on the eastern Tibetan Plateau, and the SOC pool was separated into labile C pool I (LOC I), labile C pool II (LOC II), and recalcitrant C pool (ROC) in order to investigate changes in contents of SOC fractions that have different biochemical stabilities after the establishment of cultivated grassland.

**Results:**

Although the establishment of cultivated grasslands led to increases in soil total organic C content, the increase was only significant in samples with 17 years of cultivation. We found that the contents of the three SOC fractions were higher at CG3 and CG12 compared with those in the DM, and the differences were only significant for soil LOC II. By comparison, 17 years of cultivation led to significant increases in all of the SOC fraction contents. The results implied that different cultivation years had distinct impacts on SOC fractions in cultivated grasslands, and longer cultivation years contributed to accumulated soil ROC. The recalcitrance index of SOC in the DM was higher than that at CG3 and CG12, but lower than that at CG17. This was possibly due to the generally low litter quality of cultivated grasslands, which led to a slow release of complex compounds to soils. Moreover, it was observed that soil C:N ratio was a potential indicator of SOC biochemical stability because of their close correlation.

**Conclusions:**

Our findings suggest that the long-term establishment of cultivated grasslands on DM is a promising solution to recovering both the quantity and stability of SOC on the Tibetan Plateau.

## Introduction

Grasslands, one of the most widespread terrestrial ecosystems, cover approximately one-fifth of the global land surface and play a key role in the terrestrial carbon (C) cycle (Schiedung et al., 2019; Gomez-Casanovas et al., 2021). Grassland soils are a potential sink of atmospheric C and contribute 10–30% of the global soil organic C (SOC) stock (Follett & Reed, 2010). Unfortunately, it is estimated that nearly half of the global grasslands have been degraded to some extent, leading to substantial losses of the SOC pool (Dlamini, Chivenge & Chaplot, 2016; Bardgett et al., 2021). The decline of the SOC stock in grasslands poses enormous threats to both climate regulation and food security (Bengtsson et al., 2019). Therefore, recovering the SOC level has become a challenge in the sustainable development of grasslands.

Globally, sowing seed mixtures is one of the most frequently-used methods for restoring degraded grasslands (Török et al., 2011). Although the dynamics of the SOC pool after establishing cultivated grasslands have been well-documented, the results are inconsistent due to differences in cultivation years or site conditions across individual studies (Wu et al., 2010; Dong et al., 2012; Crème et al., 2016). Moreover, most studies only took the quantity of SOC into consideration (Teixeira et al., 2011; Li et al., 2019), leaving the change in SOC stability after establishing cultivated grasslands inadequately addressed. In general, there are three principal mechanisms that stabilize SOC: physical protection by soil aggregates, chemical protection by organo-mineral complexes, and biochemical protection through chemical recalcitrance of organic molecules (Zeraatpishe & Khormali, 2012; Zeraatpisheh et al., 2021). Since soil organic matter is a complex entity that is comprised of various kinds of compounds (Lehmann & Kleber, 2015), the proportion of recalcitrant C in the total organic C (TOC) pool therefore determines the biochemical stability of SOC (Zeraatpishe & Khormali, 2012; Zeraatpisheh et al., 2021). Using the acid hydrolysis approach, SOC can be separated into a labile C pool and a recalcitrant C pool, the latter representing almost all stable SOC components such as lignin, wax, fat, suberin, and resin (Rovira & Vallejo, 2002). Some studies have applied this method to evaluate the changing biochemical stability of SOC following the ecological restoration of degraded ecosystems. For example, Zhang et al. (2022) observed that 160 years of natural vegetation restoration improved the proportion of recalcitrant C content in the TOC pool of degraded croplands on the Loess Plateau of northwest China. Shifting quality of plant litter, dead roots, or root exudates, which is often indicated by their C to nitrogen (N) ratio, is the most likely cause of the changing biochemical stability of SOC after vegetation restoration (Cheng et al., 2013; Castellano et al., 2015). Although low-quality (high C:N ratio) plant litters generally have a lower C accumulation rate than high-quality (low C:N ratio) plant litters, most of the C derived from low-quality litters has difficulty being decomposed and can stabilize in the mineral soil matrix (Castellano et al., 2015; Crème et al., 2016). Empirical evidence has shown that the establishment of cultivated grassland can greatly affect the composition of the plant community (Wang et al., 2013), litter quality, and SOC fractions that have different biochemical stabilities. However, temporal variation in the biochemical stability of SOC, as well as its influence after the establishment of cultivated grasslands, remain unclear and require more evidence to clarify.

The Tibetan Plateau covers more than 2.5 million km^2^ and is known as the earth’s “Third Pole” (Yao et al., 2012). Alpine meadows, which are dominated by *Kobresia* (Cyperaceae) species and provide a variety of ecosystem services such as C sequestration and food supply, are a major vegetation type on the Tibetan Plateau (Li et al., 2013). Nevertheless, over 90% of the alpine meadows on the Tibetan Plateau have been degraded as a result of overgrazing, climate change, and rodent activity since the 1980s (Harris, 2010). Human intervention in the establishment of cultivated grassland is a commonly-used technique for rehabilitating heavily degraded alpine meadows in this area (Li et al., 2013). Previous studies have used SOC content or stock as an indicator when evaluating the restoration effect of cultivated grasslands on the Tibetan Plateau. Although most studies suggested that establishing cultivated grasslands on degraded alpine meadows contributed to SOC accumulation (Wu et al., 2010; Su et al., 2015), declines in SOC levels after the establishment of cultivated grasslands were also reported (Shi et al., 2010; Dong et al., 2012). In general, SOC dynamics are controlled by the inputs (*e.g.*, litter) and outputs (*e.g.*, decomposition) of soil organic matter, and the chemical recalcitrance of organic molecules is considered a vital factor affecting the outputs of soil organic matter (Zeraatpishe & Khormali, 2012; Paul, 2016; Zeraatpisheh et al., 2021). Empirical evidence has shown that grasses such as *Elymus nutans* and *Poa crymophila*, which are dominant species in the cultivated grasslands on the Tibetan Plateau, have higher C:N ratios than other plant functional groups (Yang et al., 2021). Hence, the decomposition process of plant litter in cultivated grasslands may be different from that in natural grasslands, causing changes in the biochemical stability of SOC. However, this speculation needs further studies to verify. In this study, a heavily degraded alpine meadow and cultivated grasslands established for different years on the eastern Tibetan Plateau were selected. The main objectives of this study were to: (1) investigate the impacts of establishing cultivated grassland on SOC fractions that have different biochemical stabilities; and (2) explore the influencing factors that control the biochemical stability of SOC. We hypothesized that cultivated grasslands with different cultivation years have distinct impacts on the biochemical stability of SOC because the decomposition of low-quality plant litters is a slow process.

## Materials & Methods

### Study area

The study was carried out at the Sanjiangyuan Grassland Ecosystem National Observation and Research Station (34°47′N, 100°12′E, a.s.l. 3753 m) in Maqin County, Golog Tibetan Autonomous Prefecture, Qinghai Province on the eastern Tibetan Plateau. The region has a typical highland continental climate with a mean annual air temperature of −0.6 °C. The mean annual precipitation is 513 mm, mainly occurring between May and September. The annual evaporation and solar radiation levels are 1,459 mm and 2,571 h, respectively. There is no entirely frost-free period. The vegetation in this region is alpine meadow, dominated by *Kobresia pygmaea*, *Kobresia humilis*, and *Kobresia capillifolia*. The soil is classified as alpine meadow soil according to the Chinese Soil Classification System, or as Cryrendoll in the USDA Soil Taxonomy System with a shallow soil layer (<40 cm) and a sandy loam texture (Li et al., 2014).

### Site selection and field sampling

In this study, we selected a heavily degraded alpine meadow (DM) dominated by *Gentiana macrophylla* and *Elsholtzia densa* and three adjacent cultivated grasslands established for 3 years (CG3), 12 years (CG12), and 17 years (CG17) to investigate the changes in SOC fractions over the restoration time (Fig. S1). The study area covers approximately 1.8 ha. The three cultivated grasslands were all converted from DM and established with *Elymus nutans*, *Poa crymophila*, and *Festuca sinensis* (the sowing ratio was 2:1:1). (NH_4_)_2_HPO_4_ was applied at a rate of 45 kg ha^−1^ to guarantee the nutrient supply for the growth of plant seedings. Although livestock grazing had been ceased due to the poor growth of plant community in the DM, rodent activity (*e.g.*, burrowing) was still out of control. Grazing activity ceased during the warm season at the three cultivated grasslands, all of which were subjected to light grazing during the cold season. Field sampling was conducted in August 2021. At each site, five 0. 5 × 0.5 m quadrats were chosen to harvest the above-ground plant community biomass. Five soil cores (Φ: 7 cm) were randomly collected from the 0–20 cm soil layer in each quadrat and then mixed to measure the root biomass of the plant community. Soil samples used for chemical analysis were collected using the same sampling procedure of the root biomass. A total of 20 composite soil samples (four sampling sites × five quadrats) were thus obtained. In addition, an intact soil core (100 cm^3^) was sampled in each quadrat to detect the soil bulk density (BD). The collected soil samples were separated into two sub-samples. One was air-dried and sieved (two mm or 0.25 mm) to measure soil pH, particle size, and the contents of the SOC fractions and total N (TN). The other sub-sample was stored at 4 °C before determining the content of soil microbial biomass C (MBC). The soil properties of each site are presented in Table 1. The plant samples were powdered using a pulverizer after drying at 60 °C to measure their C and N content.

**Table 1:** Soil bulk density (BD), pH, microbial biomass carbon (MBC) content and particle size distributions (mean ± standard deviation) at each site. Different uppercase letters indicate the significant differences among sites (*p* < 0.05).

**Site**	**BD** **(g kg** ^−1^ **)**	**pH**	**MBC content** **(mg kg** ^−1^ **)**	**Particle size (%)**		
				**Clay**	**Silt**	**Sand**
DM	1.33 ± 0.05A	8.2 ± 0.1A	596.6 ± 222.9B	6.2 ± 0.6A	57.4 ± 1.6A	36.4 ± 1.4B
CG3	1.37 ± 0.07A	7.7 ± 0.3B	794.6 ± 242.7B	5.6 ± 0.3A	54.3 ± 3.7AB	40.0 ± 3.7AB
CG12	1.45 ± 0.12A	8.1 ± 0.1A	1074.8 ± 162.0A	5.9 ± 1.2A	49.9 ± 5.9B	44.2 ± 4.9A
CG17	1.36 ± 0.09A	7.7 ± 0.5B	1244.3 ± 189.4A	6.8 ± 1.3A	53.9 ± 4.6AB	39.4 ± 4.1AB

### Laboratory analysis

The plant samples were washed and dried at 60 °C to determine their dry weight. Soil pH was detected using a SevenEasy pH meter (Mettler-Toledo, Greifensee, Switzerland) at a 1:5 (*w*:*v*) soil to water ratio. Soil particle size distribution was analyzed using a BT-2001 laser diffraction particle analyzer (Baite, Dandong, China). Soil MBC content was determined based on the CHCl_3_ fumigation-K_2_SO_4_ extraction method (1:4, *w*:*v*). The two-step acid hydrolysis method proposed by Rovira & Vallejo (2002) was adopted to divide the SOC pool into labile pool I (LOC I), labile pool II (LOC II), and recalcitrant pool (ROC). Briefly, 0.5−1.0 g of the sieved soil sample (0.25 mm) was successively hydrolyzed with 20 mL of 5 N H_2_SO_4_ at 105 °C for 30 min, 2 mL of 26 N H_2_SO_4_ at room temperature overnight, and 2 N H_2_SO_4_ at 105 °C for 3 h in sealed Pyrex tubes. The hydrolysate and residue were separated by centrifugation and decantation after each hydrolysis. The hydrolysates generated by the first and third hydrolysis were used for determining the contents of LOC I and LOC II, respectively, while the remaining residue after three hydrolyzes was washed with deionized water and dried at 60 °C to measure the content of ROC. The contents of LOC I and LOC II were analyzed using an OI Analytical Aurora model 1030 TOC analyzer (OI Analytical, College Station, TX, USA), whereas the ROC content was determined using to the H_2_SO_4_-K_2_Cr_2_O_7_ oxidation method (Liu et al., 2020). The soil TN and plant N contents were analyzed using a FOSS Kjeltec™ 8400 Analyzer Unit (FOSS, Hillerød, Denmark) based on the Kjeldahl method. A Vario MICRO cube elemental analyzer (Elementar, Hanau, Germany) was employed to determine plant C content.

### Calculation and statistical analysis

Soil TOC content was calculated by adding the contents of LOC I, LOC II, and ROC together. The soil and plant C:N ratios were computed on a mass basis. The recalcitrance index of SOC (RIC) was obtained according to the following equation to reflect the proportion of ROC content in the TOC pool:

(1) \begin{eqnarray*}RIC(\text{%})= \frac{{C}_{ROC}}{{C}_{TOC}} \times 100\text{%}\end{eqnarray*}

where *C*_*ROC*_ (g kg^−1^) and *C*_*TOC*_ (g kg^−1^) are the contents of ROC and TOC, respectively.

All data were examined for normality and homogeneity of variance, and the results indicated that the data conformed to a normal distribution and had equality of variance. One-way analysis of variance (ANOVA) with a least significant difference (LSD) *post hoc* test was performed to examine the differences in plant characteristics and soil properties across the sampling sites. We used regression analysis to analyze the relationships between the contents of LOC I, LOC II, ROC, and TOC and those between RIC, soil C:N ratio, and plant C:N ratio. All the statistical analyses were performed using SPSS 22.0 (SPSS Inc., Chicago, IL, USA). Figures were drawn using OriginPro 9.0 (Originlab Inc., Northampton, MA, USA).

## Results

### Above-ground and root biomass of plant community

**Figure 1:**
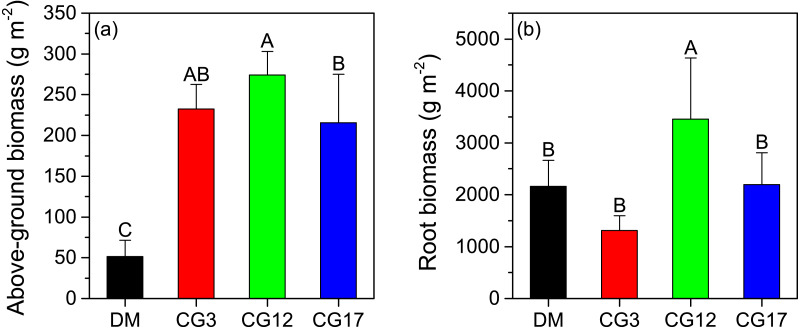
Above-ground (A) and root biomass (B) at each site. Error bars are standard deviations of the means. Different uppercase letters indicate the significant differences among sites (*p* < 0.05).

As shown in Fig. 1A, the above-ground biomass of the plant community in the DM was 51.6 g m^−2^, which was significantly lower than that of the cultivated grasslands (*p* < 0.05). The plant above-ground biomass at CG3, CG12, and CG17 were 232.2 g m^−2^, 274.1 g m^−2^, and 215.8 g m^−2^, respectively, and a significant difference was detected between CG12 and CG17 (*p* < 0.05). The root biomass of the plant community varied from 1,312.9 g m^−2^ to 3,458.9 g m^−2^ at different sites. The highest root biomass was observed at CG12, which was significantly higher than those at other sites (*p* < 0.05). By contrast, the difference in root biomass among DM, CG3, and CG17 was not significant (*p* > 0.05) (Fig. 1B).

### TOC content, TN content, and C:N ratio of soil

**Figure 2:**
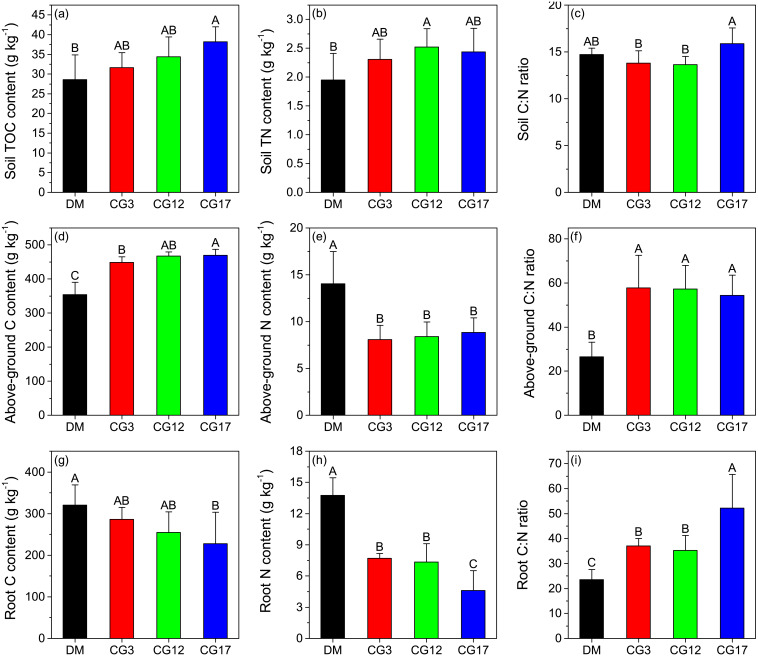
Carbon content, nitrogen content, and C:N ratio of soil (A–C), above-ground plant (D–F), and root (G–I). TOC and TN represent total organic C and total N, respectively. Error bars are standard deviations of the means. Different uppercase letters indicate the significant differences among sites (*p* < 0.05).

The soil TOC content was 28.6 g kg^−1^ in the DM, which was 9.6–25.2% lower compared to that of the cultivated grasslands. However, a significant difference in soil TOC content was only detected between DM and CG17 (*p* < 0.05). Although the soil TOC content showed an increasing trend with cultivation years, the changes were not significant (*p* > 0.05) (Fig. 2A). Compared to that of the DM, the soil TN content increased by 18.5–29.2% after establishing cultivated grasslands. Similar to soil TOC content, soil TN content also did not differ significantly among the three cultivated grasslands (*p* > 0.05) (Fig. 2B). Both CG3 and CG12 had lower soil C:N ratios than DM, whereas no significant difference was found among them (*p* > 0.05). The highest soil C:N ratio among the four sites was 15.9 at CG17, and this ratio was significantly higher than those at CG3 and CG17 (*p* < 0.05) (Fig. 2C).

### C content, N content, and C:N ratio of plant

The C content of above-ground plants varied between 354.6–469.6 g kg^−1^ at different sites, and the highest and lowest contents were detected at CG17 and DM, respectively. Moreover, we found that the C contents of the above-ground plants in the cultivated grasslands were all significantly higher compared to that in the DM (*p* < 0.05) (Fig. 2D). In contrast, the N content of the DM’s above-ground plants was significantly higher than that of the three cultivated grasslands (*p* < 0.05). Similar to the C content, the N content of the above-ground plant also showed an increasing trend with cultivation years, although generally low and not significant (*p* > 0.05) (Fig. 2E). As shown in Fig. 2F, significant increases in the C:N ratio of the above-ground plant were observed after the establishment of cultivated grasslands. The C:N ratios of the above-ground plants in the three cultivated grasslands were 2.1−2.2 times higher than that in the DM. The root’s C content was lower than that of the above-ground plants at each site. After establishing cultivated grasslands, the root’s C content decreased by 10.9–29.1%, but the decrease was only significant at CG17 (*p* < 0.05) (Fig. 2G). Similarly, the establishment of cultivated grasslands also led to significant decreases in root N content (*p* < 0.05). The highest reduction rate was -66.6%, which was observed after 17 years of cultivation (Fig. 2H). In contrast to C and N content, the root C:N ratios significantly increased by 49.9–122.0% after establishing cultivated grasslands (*p* < 0.05). The C:N ratios of root were 37.0, 35.3, and 52.2 at CG3, CG12, and CG17, respectively, and significant differences were detected between CG17 and the other two sites (*p* < 0.05) (Fig. 2I).

### SOC fraction and RIC content

**Figure 3:**
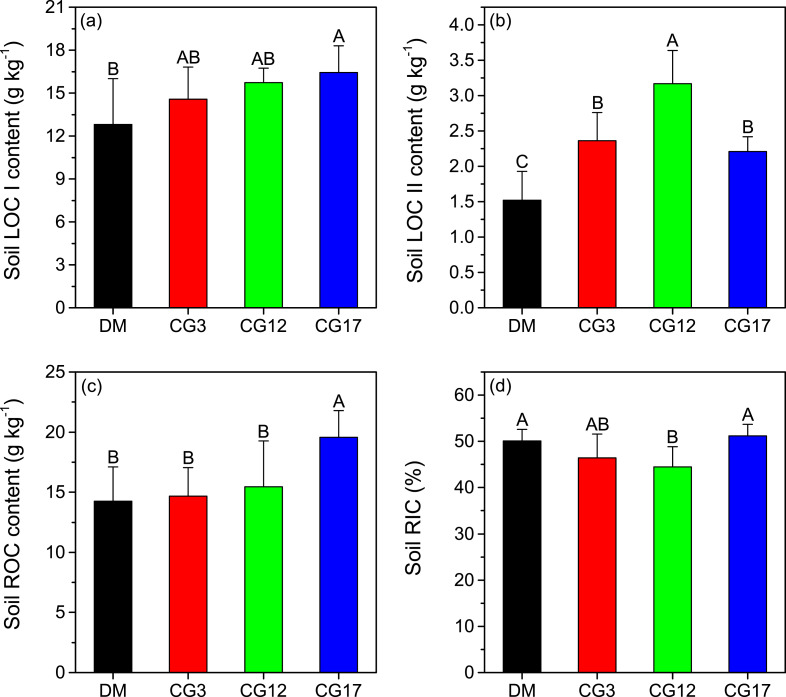
Contents of soil labile carbon pool I (LOC I) (A), labile carbon pool II (LOC II) (B), recalcitrant carbon pool (ROC) (C), as well as the recalcitrance index of soil organic carbon (RIC) (D) at each site. Error bars are standard deviations of the means. Different uppercase letters indicate the significant differences among sites (*p* < 0.05).

The soil LOC I contents varied from 12.8 g kg^−1^ to 16.4 g kg^−1^ at different sites. The highest soil LOC I content of the different sites was detected at CG17, which was significantly higher compared to that in the DM (*p* <0.05) (Fig. 3A). Soil LOC II contents were considerably lower than soil LOC I contents across all the sites. After establishment of cultivated grasslands, soil LOC II contents significantly increased by 45.4–108.6% (*p* < 0.05), and the highest increase was found at CG12 (Fig. 3B). As illustrated in Fig. 3C, there was no significant difference in the content of ROC across DM, CG3, and CG12 (*p* > 0.05). By contrast, the content of ROC at CG17 was significantly higher than those at other sites (*p* < 0.05). Soil RIC was 50.1%, 46.4%, 44.5%, and 51.2% at DM, CG3, CG12, and CG17, respectively. We found that 12 years of cultivation resulted in a significant reduction in soil RIC (*p* < 0.05). Thereafter, soil RIC showed a significant increasing trend (*p* < 0.05), but the difference in soil RIC between DM and CG17 was not significant (*p* > 0.05).

### Relationships across LOC I, LOC II, ROC, and TOC content

**Figure 4:**
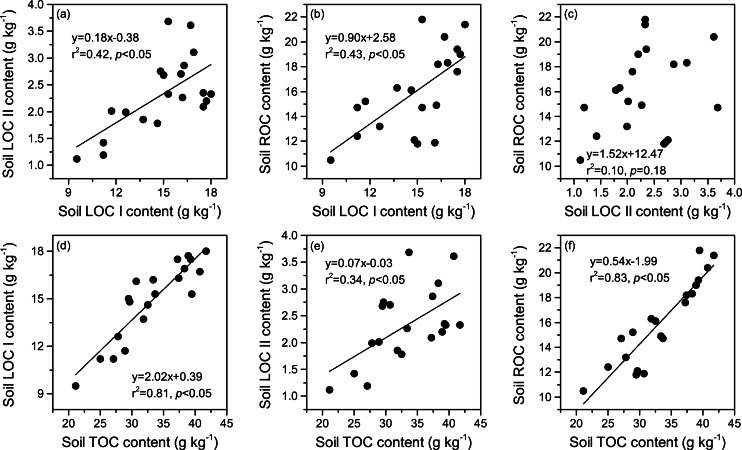
(A–F) Relationships between the contents of soil labile carbon pool I (LOC I), labile carbon pool II (LOC II), recalcitrant carbon pool (ROC), and total organic carbon (TOC).

The relationships between soil LOC I, LOC II, ROC, and TOC contents are illustrated in Fig. 4. We found that the contents of LOC I were positively correlated to those of both LOC II and ROC (*p* < 0.05). Nevertheless, there was no significant relationship between the contents of LOC II and ROC (*p* > 0.05). Significantly positive correlations were also observed between the contents of TOC and those of each SOC fraction (*p* < 0.05), and the highest correlation was found between the TOC and ROC (*r*^2^ = 0.83, *p* < 0.05).

## Discussion

### Change in soil TOC content after establishment of cultivated grasslands

Alpine meadows on the Tibetan Plateau contain approximately 16.3 Pg of SOC, which accounts for 11.4% of the total SOC storage in China (Genxu et al., 2002). Unfortunately, the degradation of alpine meadows in this region has induced substantial losses of SOC in recent decades (Wen et al., 2013). For instance, the results of our previous meta-analysis indicated that the degradation of alpine meadows on the Tibetan Plateau reduced SOC stock by an average of 51.0% (Liu et al., 2021). In the present study, it was found that soil TOC contents in the cultivated grasslands were 10.7–33.8% higher compared to that in the DM (Fig. 2A), indicating that the establishment of cultivated grasslands on DM contributed to the recovery of the SOC pool. The increased soil organic matter inputs through plant litter, root exudates, and microbial necromass after establishing cultivated grasslands was a likely reason for the enhanced SOC level (Zimmermann, Dauber & Jones, 2012; Liang et al., 2019) because the biomass of both the plant community and soil microorganisms was generally higher in the cultivated grasslands than in the DM. Moreover, the low grazing intensity in the cultivated grasslands may provide favorable conditions for the formation of soil aggregates, which can provide physical protection for SOC (Wang et al., 2020). Since soil N has been recognized as a key element limiting the formation of soil organic matter (Van Groenigen et al., 2017), the increased soil N content (Fig. 2B) also contributes to SOC sequestration after converting DM to cultivated grasslands (Fornara & Tilman, 2012). However, we found that the increase in soil TOC content was only significant in samples with 17 years of cultivation (Fig. 2A). Similarly, Wen et al. (2018) reported that soil TOC stock in the 0–60 cm soil layer gradually increased after converting a DM to cultivated grassland on the northeast edge of the Tibetan Plateau. Their results also showed that only cultivated grassland with many years of cultivation (16 years) had a higher soil TOC stock than the DM. In other regions of the Tibetan Plateau, some studies observed that TOC stock in topsoil decreased after the establishment of cultivated grasslands in the first several years, and gradually increased thereafter (Wu et al., 2010; Su et al., 2015). These findings imply that the recovery of the SOC pool is generally a slow process in the alpine region, probably as a result of the limited soil humification in the low temperature environment (Xu et al., 2015). Consequently, it is suggested that a long-term establishment of cultivated grasslands on DM is a promising solution to recover SOC levels on the Tibetan Plateau.

### Contributions of each SOC fraction to soil TOC pool

Chemical recalcitrance of organic molecules has been identified as one of the most important mechanisms that stabilize SOC (Zeraatpishe & Khormali, 2012; Zeraatpisheh et al., 2021). The SOC fractions derived from using the acid hydrolysis method are effective indicators reflecting the biochemical stability of SOC (Rovira & Vallejo, 2002). In general, LOC I is composed of non-cellulosic polysaccharide, which comes from either plant or microbial residues. LOC II mainly includes cellulose, whereas the major compounds of ROC are lignin, wax, fat, suberin, resin, and humic substances (Rovira & Vallejo, 2002; Ding et al., 2012). Due to its high resistance to decomposition, the ROC pool represents nearly all stable SOC components and plays a key role in SOC sequestration (Zeraatpishe & Khormali, 2012; Zeraatpisheh et al., 2021). In this study, we found that the proportion of ROC in TOC reached 44.5–51.2% in the top 20 cm soil layer (Fig. 2D), indicating that approximately half of the TOC pool was biochemically protected in this region. The proportion was similar to the one reported by Paul, Collins & Leavitt (2001), who found that ROC accounted for 33.0–65.0% of the TOC pool in agricultural soils in the midwestern US. In northern Tibet, Guan et al. (2018) reported that alkyl C and aromatic C, both of which consisted of compounds that difficult to be decomposed, were essential chemical compositions (46.5–48.3%) of SOC in an alpine meadow. Xiao et al. (2021) observed a relatively lower proportion of alkyl C (30.7%) and aromatic C (5.7%) in the TOC pool in an alpine meadow on the northeastern Tibetan Plateau. By comparison, some studies found higher proportions of ROC in TOC (>85%) in temperate and subtropical grasslands (Belay-Tedla et al., 2009; Feyissa et al., 2020). Hence, ROC may occupy a relatively lower proportion in TOC in the alpine grasslands of the Tibetan Plateau. Similar to the observations of previous studies (Ding et al., 2012; Liu et al., 2020), our results also indicated that soil labile C content generally had positive relationships with soil ROC content (Figs. 4A–4C). This was attributed to the fact that the proportions of SOC fractions in TOC were relatively stable in a small research scale, and had similar climatic conditions, parent material, and soil type (Belay-Tedla et al., 2009). Moreover, significantly positive relationships were also detected between TOC and each SOC fraction, and the highest correlation was observed between TOC and ROC (Figs. 4D–4F). Given the high chemical recalcitrance and content of ROC, it is suggested that recalcitrant C pool plays a vital role in controlling both the quantity and stability of SOC during the artificial restoration of DM on the Tibetan Plateau.

**Figure 5:**
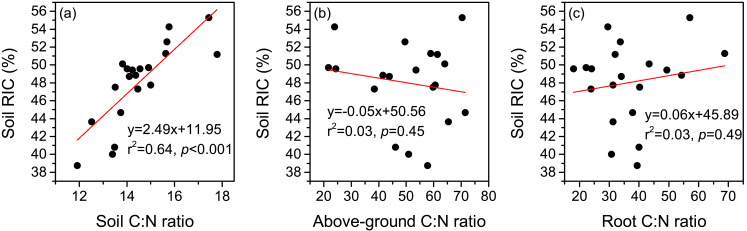
Relationships between the recalcitrance index of soil organic carbon (RIC), and C:N ratios of soil (A), above-ground plant (B), and root (C).

### Changes in SOC fractions after the establishment of cultivated grasslands

Land-use change has been recognized as a critical factor affecting both the inputs and outputs of SOC (Ramesh et al., 2019). As a consequence, the biochemical stability of SOC may vary over time after the conversion of land-use types (Cheng et al., 2013; Zhang, Wang & Wang, 2014). For instance, Cheng et al. (2013) found that the proportion of ROC in TOC showed an increasing trend after 15 years of afforestation in the Danjiangkou Reservoir area of subtropical China, which they mainly attributed to higher inputs of low-quality plant litter. The establishment of cultivated grassland, which completely changes the composition of the plant community, is a widely-used method to restore degraded alpine grasslands on the Tibetan Plateau (Wang et al., 2013). However, the change in SOC stability after the establishment of cultivated grasslands in this region remains unclear. In this study, we found that cultivated grasslands with 3 years and 12 years of cultivation showed increased contents of soil LOC I and LOC II, with a limited impact on the content of soil ROC (Figs. 3A–3C). Such changes resulted in declines in soil RIC (Fig. 3D). Therefore, soil labile C pool may play a more important role in SOC sequestration than the recalcitrant C pool when the cultivation years are less than 12 years. However, it should be noted that the accumulated labile C pool may be rapidly lost due to its low chemical recalcitrance corresponding with external environment changes (*e.g.*, global warming). In contrast, the contents of all the SOC fractions significantly increased after 17 years of cultivation, and soil RIC at CG17 was similar to that in the DM (Fig. 3). The results were in line with our hypothesis that different cultivation years have distinct impacts on the biochemical stability of SOC in cultivated grasslands. Similar to our findings, Dou et al. (2013) pointed out that the reforestation of *Pinus massoniana* did not influence the content of soil ROC in the first 10 years. Afterwards, the content of soil ROC showed an increasing trend in south China. As shown in Figs. 2F and 2I, the C:N ratios of both above-ground plants and roots were significantly higher in the cultivated grasslands than in the DM, indicating declines in the quality of plant litter after establishing cultivated grasslands. As a consequence, a slow decomposition rate of plant litter in cultivated grasslands can be expected (Naeem et al., 2021). During the decomposition of plant litter, compounds with simple chemical structures (*e.g.*, cellulose) will be first decomposed by soil microorganisms (Berg & McClaugherty, 2014), while the decomposition of complex compounds (*e.g.*, lignin) is a long process, especially in the alpine environment (Wang et al., 2018). In the alpine meadow of the eastern Tibetan Plateau, a recent study observed that the longest decomposition time of grasses was 11.18 years, which was considerably longer than those of other functional plant groups (Yang et al., 2021). In this case, longer cultivation years may lead to a better decomposition of plant litter and the release of more complex compounds into soils, contributing to accumulations in soil ROC pool. As illustrated in Fig. 5, a significant positive correlation was detected between soil C:N ratio and soil RIC, suggesting that soil C:N ratio is a potential indicator that reflects the change in biochemical stability of SOC. However, the increase in soil C:N ratio also implies a restricted decomposition of plant litter (Finn et al., 2015; Hou et al., 2021). Therefore, we speculated that the accumulation of soil ROC may slow down if the soil C:N ratio continuously increases with cultivation years, which needs more evidence to verify.

## Conclusion

The observations of this study showed that the establishment of cultivated grasslands on a DM led to increases in soil TOC content, but the increase was only significant in samples with 17 years of cultivation. Across different sites, the contents of LOC I, LOC II, and ROC varied in ranges of 12.8–16.4 g kg^−1^, 1.5−3.2 g kg^−1^, and 14.3–19.6 g kg^−1^, respectively. Although the contents of the three SOC fractions were higher at CG3 and CG12 than in the DM, the differences were only significant for soil LOC II. In contrast, 17 years of cultivation led to significant increases in the contents of all the SOC fractions. The results indicated that different cultivation years had distinct impacts on SOC fractions with different biochemical stabilities, and longer cultivation years contributed to accumulations in soil ROC. Soil RIC in the DM was higher than that at CG3 and CG12 but lower than at CG17. The decreased litter quality, which was reflected by the high plant C:N ratio, was a likely reason for the changes in soil RIC after establishing cultivated grasslands. We also observed that soil RIC was positively correlated to soil C:N ratio, implying that soil C:N ratio was a potential indicator of SOC biochemical stability in the study area. In conclusion, it is suggested that long-term establishment of cultivated grasslands on degraded alpine meadows is a promising solution to recover both the quantity and stability of SOC on the Tibetan Plateau.

##  Supplemental Information

10.7717/peerj.14012/supp-1 Supplemental Information 1 Photographs of degraded alpine meadow (a) and cultivated grassland (b: 3 years; c: 12 years; and c: 17 years) selected in this study. (Photo credit: Xiang Liu)

10.7717/peerj.14012/supp-2 Supplemental Information 2 Raw data of this study

## Additional Information and Declarations

### Data Availability

The following information was supplied regarding data availability: The raw measurements are available in the Supplemental File.
