# Supplementary figures and images for: Effects of establishing cultivated grassland on soil organic carbon fractions in a degraded alpine meadow on the Tibetan Plateau
Source: PeerJ. 2022 Sep 13;10:e14012. doi: 10.7717/peerj.14012 (PMC9480066; doi:10.7717/peerj.14012)

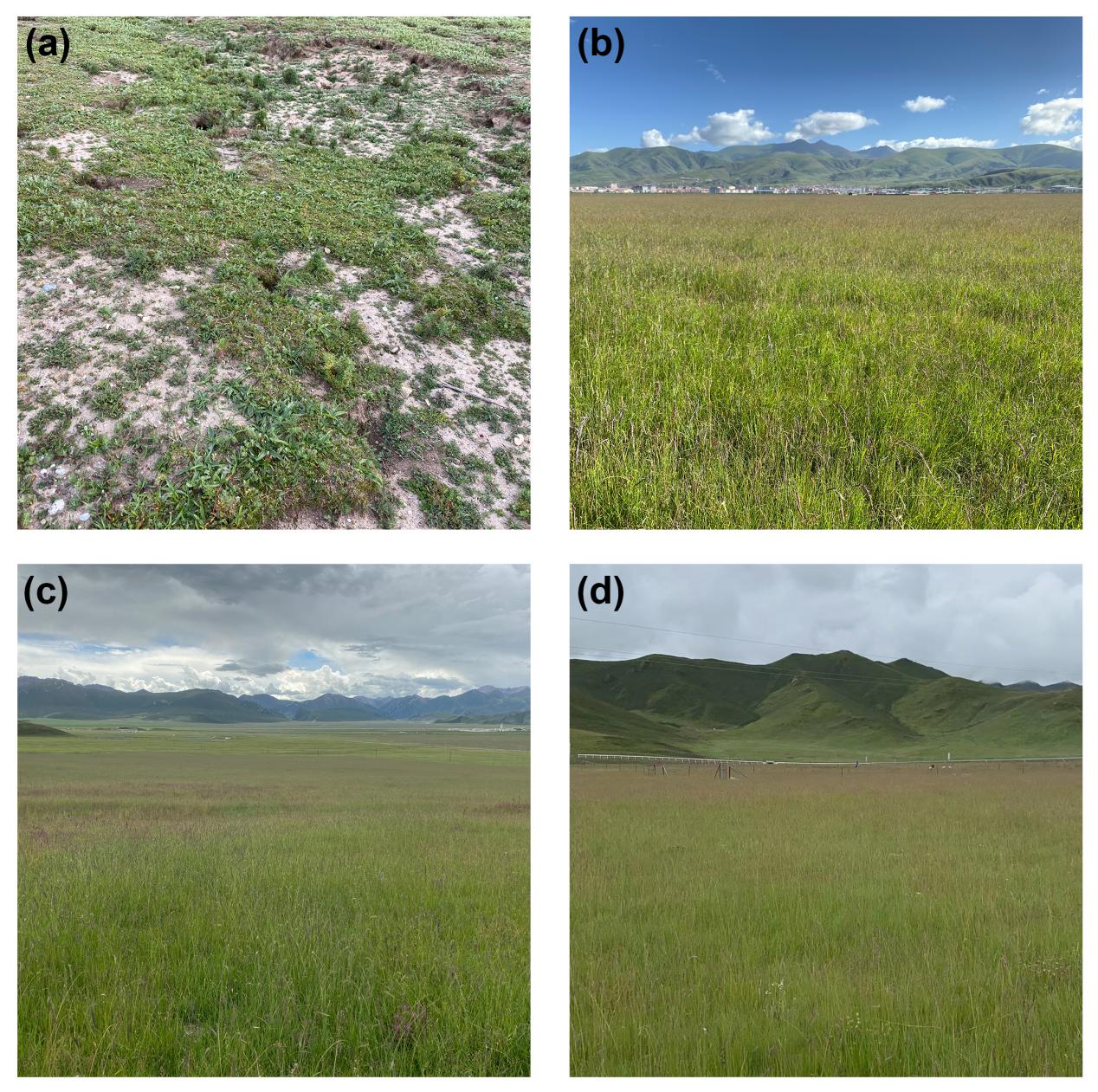

Supplement: Supplemental Information 1 [file peerj-10-14012-s001.jpg]
